# Macroecological Evidence for Competitive Regional-Scale Interactions between the Two Major Clades of Mammal Carnivores (Feliformia and Caniformia)

**DOI:** 10.1371/journal.pone.0100553

**Published:** 2014-06-27

**Authors:** Rasmus Østergaard Pedersen, Brody Sandel, Jens-Christian Svenning

**Affiliations:** Section for Ecoinformatics & Biodiversity, Department of Bioscience, Aarhus University, Aarhus, Denmark; Università degli Studi di Napoli Federico II, Italy

## Abstract

Geographical gradients in species diversity are often explained by environmental factors such as climate and productivity. Biotic interactions play a key role in evolutionary diversification and may therefore also affect diversity patterns, but this has rarely been assessed. Here, we investigate whether negative competitive interactions shape the diversity patterns of the two major mammalian clades of carnivores, the suborders Caniformia (dogs and allies) and Feliformia (cats and allies) within the order Carnivora. We specifically test for a negative effect of feliform species richness on caniform species richness by a natural experiment, The Great American Interchange, which due to biogeographic lineage history and climate patterns caused tropical South America to be colonized by most caniform families, but only one feliform family. To this end we used regression modelling to investigate feliform and caniform richness patterns and their determinants with emphasis on contrasting the Old and New World tropics. We find that feliform richness is elevated in the Old World Tropics, while caniform richness is elevated in the New World Tropics. Models based on environmental variables alone underpredict caniform richness and overpredict feliform richness in the New World and vice versa in the Old World. We further show that models including feliform richness as a predictor for caniform species richness significantly improve predictions at the continental scale, albeit not at finer scales. Our results are consistent with a negative effect of feliforms on regional-scale caniform diversification within the tropics, probably indicating that niche space occupancy by the one clade constrains diversification in the other in the build-up of regional faunas, while negative interactions at smaller scales may be unimportant due to niche differentiation within the regional faunas.

## Introduction

Species diversity patterns are often explained only in terms of environmental factors such as climate and habitat. In evolutionary time biotic interactions play a key role in species diversification and should therefore be considered when assessing species diversity patterns, although this has rarely been done. In this article we investigate how competitive interactions amongst the Carnivora, shape the diversity patterns of its two extant subclades. The main group of carnivorous mammals is the order Carnivora [1]. The extant species of this group are split into the suborders Feliformia (cats and their relatives such as hyaenas, mongooses etc.) and Caniformia (dogs and their relatives such as bears, weasels etc.). Almost all feliforms are carnivores, as are most caniforms, although caniforms have a greater tendency towards omnivory and even include a few herbivores [1]. The terrestrial Carnivora are fairly homogeneous in general structure, constraining the two groups to similar feeding niches [2]–[4]. For example, a study of tigers (*Panthera tigris*), leopards (*Panthera pardus*) and dholes (*Cuon alpinus*) found a substantial dietary overlap between all three species [5]. Further, interspecies killing between Carnivora species is widespread, accounting for up to 68% of mortality in some species [6]. This implies some degree of competition between the two groups where they overlap, but whether such interactions affect their current geographical diversity patterns has not been assessed.

The two suborders differ in historical biogeography, providing a natural experiment for assessing the interaction of the two clades. Most feliform families have originated in the Old World, and diversified throughout Africa and the southern latitudes of Europe and Asia [7], and primarily remained in the tropics [8]. Caniformia on the other hand have also diversified outside of the tropics and took advantage of the Bering land bridge to spread throughout the northern temperate regions [3]. South America, including its large tropical region, was only colonized by the two suborders when the Great American Biotic Interchange (GABI) began from the Miocene onwards and only became fully realized with the full emergence of the Panama land-bridge roughly 3 million years ago [9], [10]. The GABI set the stage for a large-scale natural experiment on the role of feliform-caniform interactions in Carnivora diversification under differing migration histories and climates.

All the major caniform families, mostly adapted to the northern temperate climate, had immigrated to or evolved in North America and could thus readily take part in the GABI [3]. In contrast, most feliform families have not colonized North America from the Old World via the Bering land bridge [7]. This limited the number of feliform families in North America to one extant family (cats, Felidae) and the extinct Nimravidae and Barbourofelidae [7] (which went extinct before the GABI [3]), with one limited excursion of Hyaenidae (hyaenas) to North America (one specialized species in Early Pleistocene (*Chasmaporthetes ossifragus*); [11]). Hence, only one feliform family was able to take part in the Carnivoran colonization of South America. Furthermore, caniforms arrived in South America substantially before the feliforms. One species of procyonid carnivoran island hopped before the complete land closure between South America and North America 7.3 mya, and when the gap finally closed (2.6–2.4 mya) Mustelidae (weasels) and Canidae (dogs) were the first additional carnivorans to cross, while the first felids appear only to have arrived 1.8 mya [9]. As a result, opening of the Panama land bridge provided all major terrestrial caniform groups (Mustelidae, Canidae, Procyonidae (racoons), Ursidae (bears) [3], [10]) an evolutionary arena relatively free from competition with feliforms, compared to the tropical regions of the Old World. The GABI set the stage for a rapid evolutionary diversification into unoccupied niches, Ursidae with slow diversification (bradytelic), Felidae and Procyonidae with moderate diversification (horotelic), and the two remaining caniform groups, Canidae and Mustelidae, with fast diversification (tachytelic) [10]. Importantly, the smaller caniforms thereby had the opportunity to radiate into unoccupied niche space. A carnivore niche space occupied, in the Old World, by the smaller feliforms such as Viverridae (civets) and Herpestidae (mongooses) which are diverse in the warmer regions of the Old World [7], [8], [12] and never made it across the Bering land-bridge to North America.

We hypothesise that competition between the two extant suborders of Carnivora, Caniformia and Feliformia, has led to a constraint on caniform species richness in the Old World tropics. Due to the biogeographic differences in migration history between the feliform and caniform families, we hypothesise that the caniform families have experienced regional competitive release when the Panama land-bridge to South America allowed access to the warmer regions of the New World. Caniform families were set partially free from competition from their sister clade, notably as concerns the smaller carnivoran size classes, as only Felidae (the true cats) was there to co-invade South America. Overall, we predict lower feliform species richness in the New World tropics than expected from the environment due to limited family-level clade availability. As a consequence of release from competition with feliforms, we therefore also expect to see a higher caniform species richness in the New World tropics than expected from the environment. Further, we sought to test if a negative relationship between feliform and caniform richness distributions is only a continental-scale phenomenon reflecting evolutionary interactions in the build-up of regional faunas due to niche space occupancy or if they are also repeated on smaller spatial scales, reflecting more purely ecological interactions within current regional faunas.

## Materials and Methods

### (a) Data

Species range data were obtained for all species in the order Carnivora from the International Union for Conservation of Nature (IUCN) Red List of Threatened Species [13]. Original polygon range maps were resampled to a regular 5° equivalents (482 km×482 km) cell grid in a Behrmann Equal Area projection. The species were split into the two suborders Feliformia and Caniformia containing all extant terrestrial carnivorans. The largely marine families in Pinnipedia (walrus, eared seals, and true seals) were excluded. Within each cell, we calculated the total richness of each suborder.

To build models of environmental richness determinants, we selected four climatic variables, one productivity variable, one variable describing human influence and two variables describing habitat heterogeneity (Table 1). We selected variables to represent climate, habitat heterogeneity and human effects as these have previously been found to be important predictors of mammalian richness [14]. The climate variables selected were mean annual temperature (MAT), temperature seasonality (TS), mean annual precipitation (MAP) and precipitation seasonality (PS) (available from WorldClim [15]). Habitat heterogeneity was described by habitat diversity (HabDiv; Reciprocal Simpsons Diversity Index calculated from land cover types in GlobCover [16]) and elevation range (ElevRange; the altitude range in each grid cell, available from http://www2.jpl.nasa.gov/srtm/ via WorldClim [15]). Human effects were summarized with the human influence index (Human; calculated by adding 8 different influence scores, and varies from 0 no influence to 64 maximum influence, available from http://sedac.ciesin.columbia.edu/wildareas/downloads.jsp [17]). It has previously been shown that both HabDiv and ElevRange are important predictors of species richness in birds, and that productivity and precipitation both are significant predictors though with a large degree of correlation [18]. Both MAT and TS were included as climatic factors since MAT relates to overall energy input to the system, and TS indicates the stress of the environment (as shown with maximum daily temperature [18]). Elevation range, precipitation, temperature, productivity and habitat diversity have previously been shown to have somewhat overlapping individual effects [19]. Productivity was included in the form of the Normalized Difference Vegetation Index (NDVI), describing the amount of vegetation in the area (available at http://glcf.umd.edu/data/gimms/ [20] and described in [21], [22]).

**Table 1:** Environmental variables.

Environmental variables	Explanation
MAT	Mean annual temperature^1^
TS	Temperature seasonality^1^
MAP	Mean annual precipitation^1^
PS	Precipitation seasonality^1^
NDVI	Productivity^2^
Human	Human influence index^3^
HabDiv	Habitat diversity^4^ (Reciprocal Simpsons Diversity Index of land cover types)
ElevRange	Elevation range^5^ (Calculated as the 99th percentile of elevation – the 1st percentile.)

1: Climate variables was taken from (http://www.worldclim.org/) and calculated from 2.5′ resolution data,

2: (http://glcf.umd.edu/data/gimms/),

3: (http://sedac.ciesin.columbia.edu/wildareas/downloads.jsp),

4: (http://www.gofc-gold.uni-jena.de/wg_biomass/sites/globcover.php),

5: (http://www2.jpl.nasa.gov/srtm/) via worldclim.

The selected variables had some degree of multicollinearity, but our main focus was on feliform species richness as a predictor of caniform species richness and collinearity among the other predictors was therefore not important, as estimating their individual effects was not an aim.

### (b) Data analysis

The main analysis was done for the tropical belt, from 23.4° N to 23.4° S, as this is where we predict caniforms to be competitively released in the New World. Cells containing less than 50% land were excluded to avoid an area effect. To avoid island effects, Indonesia and Madagascar were also removed. The final dataset included 186 cells. All data handling and statistical testing was done using R (version 3.1.0) [23], and major packages: ‘raster’ [24], ‘maptools’ [25], ‘spdep’ [26], ‘ncf’ [27], ‘maps’ [28], and ‘ggplot2’ [29].

To improve normality of the data, the variables TS and ElevRange were log-transformed and MAP was square root transformed. All variables were then standardized to a mean of 0 and standard deviation of 1. Species richness and residuals were transformed back before plotting to improve interpretability of the figures.

Ordinary Least Squares (OLS) multiple regression models were fit for species richness separately for both suborders against the environmental variables. Caniform species richness was furthermore modelled against both environmental variables and feliform richness pattern, to test for a negative effect of feliforms.

We also fit spatial regression models that take spatial autocorrelation into account [30]. This was done, as with the OLS models, for both groups against environment and for caniforms also against both environment and feliform species richness. We used simultaneous Auto-regressive (SAR) error models [31].SAR models were fit using the R library ‘spdep’ [32]. We examined possible neighbourhood definitions to determine how effective each was at removing residual autocorrelation from model predictions. We found that a rook's case neighbourhood provided a satisfactory result (Figure S1). By accounting for spatial autocorrelation, the effects estimated by the SAR models may more reflect local effects, while effects estimated by the OLS models may more reflect broad-scale patterns [30].

For model comparison we used Akaike's Information Criterion, corrected for small sample size (AIC_c_), which compares the model explanatory power while compensating for additional model parameters [33]. For comparing models we calculate ΔAIC_ci_ = AIC_ci_ - AIC_cmin_, where a value of 0–2 indicates both models are approximately equally likely, while values >10 indicate that the model with higher AIC_c_ has almost no support at all [33].

## Results

### (a) Geographical species richness patterns

**Figure 1:**
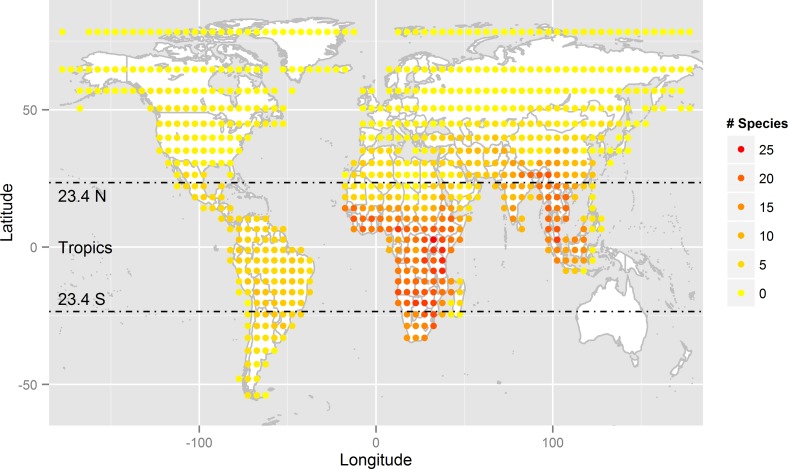
Feliform species richness distribution in a Behrmann Equal Area projection of 5° equivalents (482 km×482 km) grid size, with the tropics marked.

**Figure 2:**
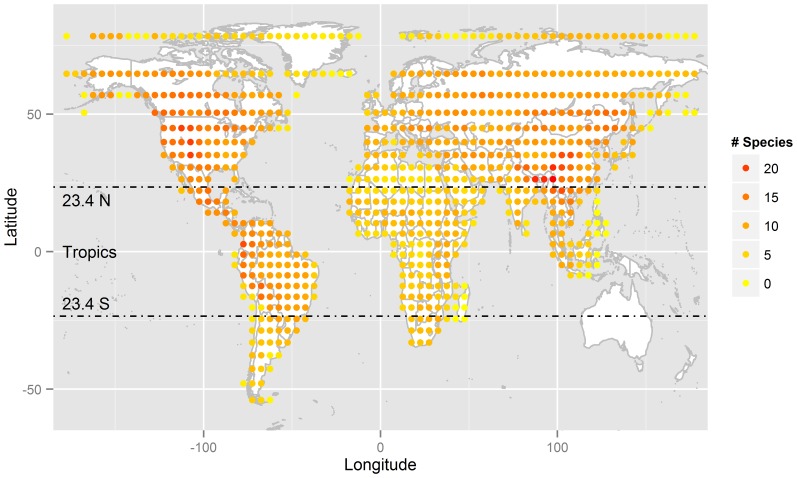
Caniformia species richness distribution in a Behrmann Equal Area projection of 5° equivalents (482 km×482 km) grid size, with the tropics marked.

**Figure 3:**
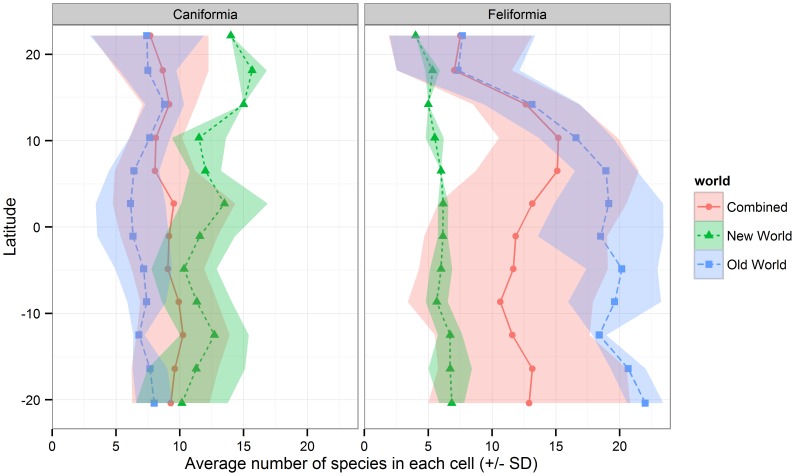
Latitudinal *Carnivora* species richness distribution. The average number of species for both Feliformia and Caniformia was calculated on each latitudinal height, in 5 degree grid-cells, for both the New World, the Old World and combined. Islands and areas with lower than 50% land was excluded.

Feliforms are concentrated in warmer regions and over-represented in Africa and southern Asia compared to the same latitude in South America (Figure 1). In contrast, caniforms are more uniformly distributed across the World (Figure 2). Within the tropical zone, feliforms are more species-rich in the Old World, while caniforms are more species-rich in the New World (Figure 3).

### (b) The effect of feliform species richness on caniform species richness

**Figure 4:**
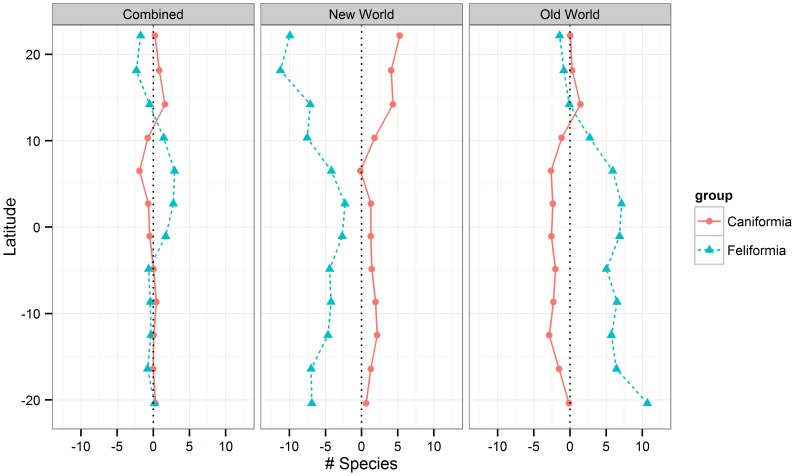
The residual number of species per cell of the ordinary least squares (OLS) models of the clade species richness against the environmental predictors. Plotted against a latitudinal gradient for both the Caniformia and Feliformia models run on all environmental predictors.

The OLS models of feliform and caniform species richness against environmental predictors (Table 2) showed a relatively consistent overprediction of feliform richness in the New World and underprediction of caniform richness (Figure 4). The opposite was the case in the Old World, where the model overpredicted caniform species and underpredicted feliform species. Adding feliform species richness to the model for caniform richness produced a substantially better model (R^2^ improvement from 0.40 to 0.51, ΔAIC_c_ = 37.8) and removed the tendency for regional over- and underpredictions.

**Table 2:** Results of the Ordinary-least-squares (OLS) multiple linear regression models of feliform and caniform species richness against environmental (ENV) predictors or these plus species richness of the competing group (Env + Feliformia).

Model	Feliformia ∼	Caniformia ∼	
	OLS: Env	OLS: Env	OLS: Env + Feliformia
MAT	−0.056	0.120	0.097
TS	−0.073	0.288**	0.258**
MAP	0.074	0.690***	0.720***
PS	−0.009	0.087	0.083
NDVI	−0.085	0.166	0.132
Human	0.023	−0.239*	−0.230**
HabDiv	0.530***	0.128	0.339***
ElevRange	−0.077	0.422***	0.391***
Feliformia	—	—	−0.398***

Ordinary-least-squares (OLS) multiple linear regression models were run for feliform species richness against all our environmental predictors (Acronyms as in Table 1), and the same was done for caniform species richness. Caniform species richness was furthermore modelled against environmental predictors and feliform species richness, showing feliform species richness as a significant predictor. Significance codes:

*** (p<0.001),

** (p<0.01) and

* (p<0.05), unmarked are not significant (p>0.05).

**Figure 5:**
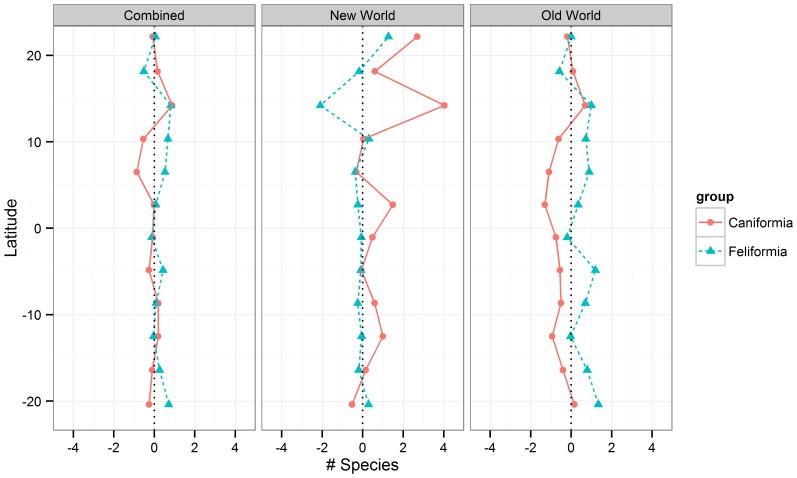
The residual number of species per cell of the spatial auto-regressive error (SAR) models of the clade species richness against the environmental predictors. Plotted against a latitudinal gradient for both the Caniformia and Feliformia models run on all environmental predictors.

The SAR models for feliform and caniform richness against environment repeated the result from the OLS models, overpredicting feliform richness and underpredicting caniform richness in the Old World (Table 3 and Figure 5), though only slightly. The SAR model of caniform richness against environment plus feliform richness did not describe the species richness distributions better than the environment alone (Table 3). When comparing the two SAR models of caniform richness, there was a ΔAIC_c_ increase of 2.3, which indicates that two models are about equally good at describing the data with a slight advantage to the model not including feliform species richness as a predictor.

**Table 3:** Results of the simultaneous auto-regressive (SAR) error models of feliform and caniform species richness against environmental (ENV) predictors or these plus species richness of the competing group (Env + Feliformia).

Model	Feliformia ∼	Caniformia ∼	
	SAR: Env	SAR: Env	SAR: Env + Feliformia
MAT	0.028	0.166*	0.165*
TS	−0.134	0.135	0.135
MAP	0.178	0.112	−0.106
PS	−0.052	0.036	0.036
NDVI	0.001	0.491*	0.494*
Human	−0.103*	−0.025	−0.023
HabDiv	0.167***	0.143*	0.140
ElevRange	0.094**	0.219***	0.217***
Feliformia	—	—	0.015

Spatial simultaneous autoregressive (SAR) error models were run for feliform species richness against all our environmental predictors (Acronyms as in Table 1), and the same was done for caniform species richness. Caniform species richness was furthermore modelled against all environmental predictors and feliform species richness, though here **not** showing feliform species richness as a significant predictor of their species richness. Significance codes:

*** (p<0.001),

** (p<0.01) and

* (p<0.05), unmarked are not significant (p>0.05).

## Discussion

Our results suggest that biogeographic history combine with biotic interactions to shape the global species richness patterns in the two main extant groups of terrestrial mammal carnivores, the suborders Caniformia and Feliformia. We found that models based on environment alone underpredict feliform species richness in the Old World tropics and overpredict it in the New World tropics, while the opposite is the case for caniform species richness. At the coarse spatial scale represented by the OLS regression models, a model additionally including feliform richness as a predictor provided stronger explanatory power of caniform richness than a model including environment alone. These patterns are consistent with the biogeographic migration history with few of the mostly low latitude [8] feliform families spreading into the New World via the high-latitude Bering land bridge, and the contingent regional competitive release of caniforms after the Panama land bridge to South America allowed access to the warmer regions of the New World. By employing the GABI as a natural experiment [10] our results point to the macro-scale applicability of micro-scale experimental results showing that immigration history and diversification are important predictors of final species composition [34]. Many carnivorans were involved in the Late Pleistocene and Holocene megafauna extinctions (Table S1). The species range data we used does not take account of how humans have influenced today's distribution of the carnivorans, opening the possibility that the patterns observed may be bias by human-driven extinctions. However, when enumerating the late Quaternary carnivoran range contractions and extinctions there is no geographic bias that could have produced our findings (Table S1, [35]–[40]). In the New World extinct caniform species outnumber extinct feliforms 11 to 6, and extinction are more equal in the Old World (3 feliforms vs. 2 caniforms) (Table S1). Hence, accounting for these extinctions would have strengthened our findings, notably with an even greater caniform richness surplus in the New World.

As discussed in a recent review, the large-scale effect of biotic interactions leading to realised species distributions have rarely been assessed [41]. His review of the literature on niche filling leads to the conclusion that phylogenetic lineages that first occupy an area tend to diversify and occupy much of the available niche space. This does not prevent the invasion of new lineages, but does tend to prevent their diversification. This again has rarely been assessed on large scale, especially for resource-related niches and species interactions [41]. Addressing this knowledge gap, our present findings of New and Old World tropical feliform and caniform discrepancies are consistent with that the introduction of all major caniform lineages to South America via the GABI, alongside only one major feliform lineage, having allowed caniforms in South America to diversify into warm environments more freely than in the Old World tropics due to reduced competition. Notably, our result directly estimate a negative effect of feliform richness on caniform richness (β = −0.398, p<0.001, Table 2). This effect could be due to interspecies competition between species in the two groups because of feeding niche overlap [2], [5], [42], including seizing killed prey from each other [43], as well as direct interspecific killing [6].

The SAR models showed similar results as the OLS models, but with a much less clear geographic difference in environmental richness residuals between the two clades, reflecting that these largely accounted for by the spatial component of the SAR models. Still, caniform richness was slightly overpredicted and feliform richness slightly underpredicted in the Old World by the environmental variables. Adding feliform richness to the SAR model for caniform richness did not clearly improve explanatory power over the purely environmental model. The discrepancy between the OLS and SAR models concerning the feliform effect on caniform species richness is consistent with the feliform effect primarily acting on the regional diversification [44] and build-up of the caniform species pool, with small-scale co-occurrence being little affected. Mammalian carnivores usually require extensive home ranges because their diet requires access to a certain amount of prey, and also tend to have large dispersal potential [1]. These traits should predispose carnivore clades to have strong influences on each other's richness at continental scales, through rapid range expansion and contingent broad-scale competitive exclusion and/or niche pre-emption constraints on evolutionary diversification [45].

The present study investigated the relationship between the geographic species richness patterns for the two major clades of terrestrial mammalian carnivores, the suborders Feliformia (cats and relatives) and Caniformia (dogs and relatives) within the order Carnivora. We find that feliform and caniform richness patterns cannot be fully explained by the environment, but instead show signatures of biogeographic history and contingent biotic interaction effects. More precisely, our findings are consistent with the biogeographic mostly low latitude migration pattern of feliform families with few migrations into the New World via the high-latitude Bering land bridge filter corridor, and the contingent regional competitive release of caniform diversification after the Panama land bridge to South America allowed access to the warmer regions of the New World. Thus, mammalian carnivores exemplify the idea that biotic interactions may indeed play a key role in shaping global-scale diversity patterns via effects on evolutionary diversification. Future studies should assess how generally important such effects are, their spatial scale dependency, and how such effects relates to geographic patterns in functional diversity and thereby community functioning.

## Supporting Information

Figure S1 **Correlogram for OLS and SAR models.** No auto-correlation was found at the α = 0.05 level after Bonferroni correction for the SAR model.
(TIFF)

Table S1 **Extinct and regionally extinct carnivorans throughout the last 130,000 years.**
(DOCX)
