# Supplementary figures and images for: Macroecological Evidence for Competitive Regional-Scale Interactions between the Two Major Clades of Mammal Carnivores (Feliformia and Caniformia)
Source: PLoS One. 2014 Jun 27;9(6):e100553. doi: 10.1371/journal.pone.0100553 (PMC4074115; doi:10.1371/journal.pone.0100553)

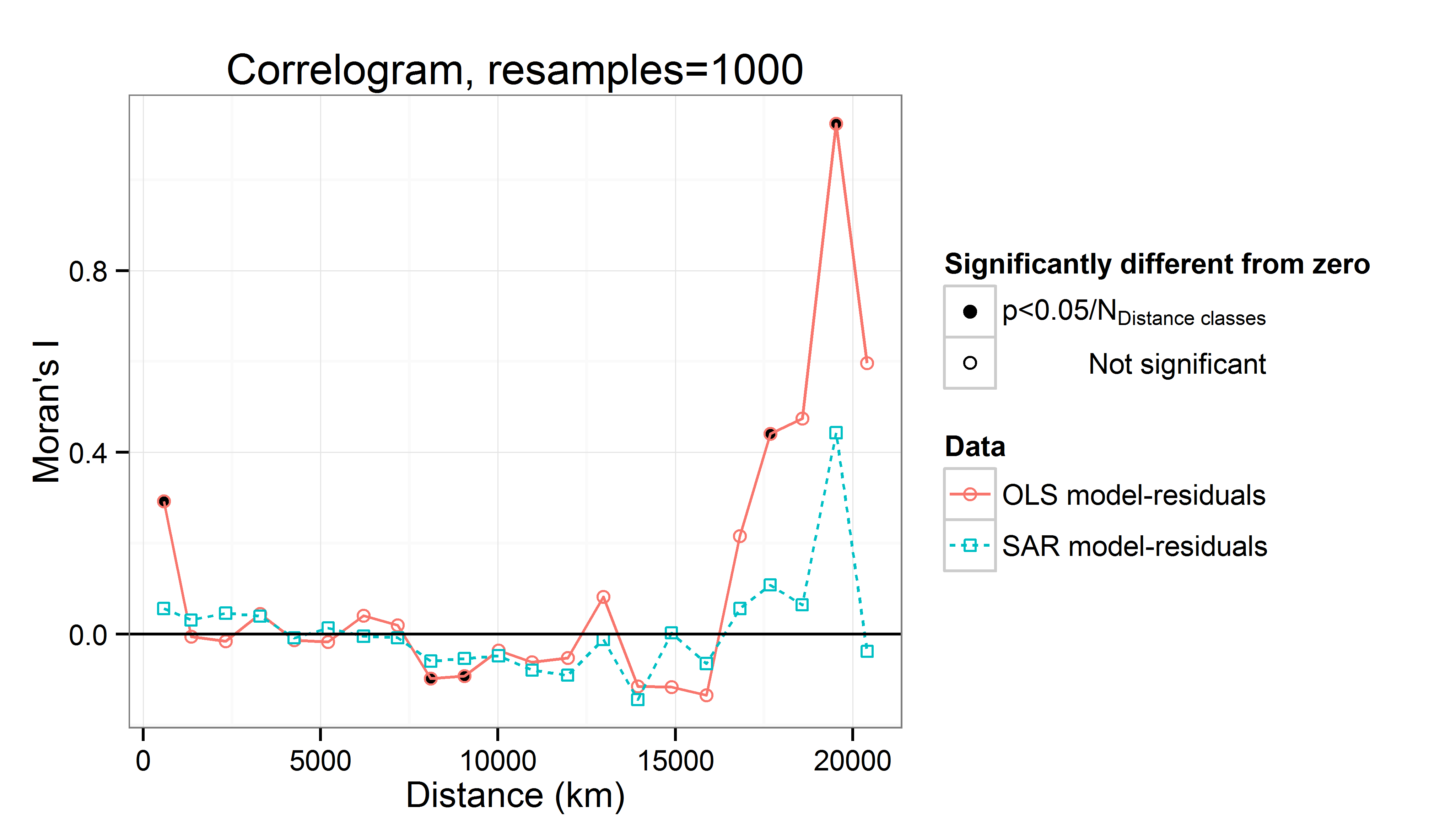

Supplement: Figure S1 — Correlogram for OLS and SAR models. No auto-correlation was found at the α = 0.05 level after Bonferroni correction for the SAR model. (TIFF) [file pone.0100553.s001.tiff]
