# Supplementary material for: Macroecological Evidence for Competitive Regional-Scale Interactions between the Two Major Clades of Mammal Carnivores (Feliformia and Caniformia)
Source: PLoS One. 2014 Jun 27;9(6):e100553. doi: 10.1371/journal.pone.0100553 (PMC4074115; doi:10.1371/journal.pone.0100553)
Supplement: Table S1 — Extinct and regionally extinct carnivorans throughout the last 130,000 years. (DOCX) [file pone.0100553.s002.docx]

**Table S2:** Extinct and regionally extinct carnivorans throughout the last 130,000 years.

| **Source** | **Clade** | **Family** | **Genus** | **Species** | **Extinction** | **Where** | **Precise** |
| --- | --- | --- | --- | --- | --- | --- | --- |
| Sandom et al. | Caniformia | CANIDAE | Canis | dirus | 130,000-1000 yBP | New World | North and South America |
| IUCN | Caniformia | CANIDAE | Dusicyon | australis | 1000 yBP-recent | New World | Falkland Islands |
| Sandom et al. | Caniformia | CANIDAE | Dusicyon | avus | 130,000-1000 yBP | New World | South America |
| Sandom et al. | Caniformia | CANIDAE | Protocyon | troglodytes | 130,000-1000 yBP | New World | South America |
| Sandom et al. | Caniformia | CANIDAE | Theriodictis | tarijensis | 130,000-1000 yBP | New World | South America |
| Sandom et al. | Caniformia | MEPHITIDAE | Brachyprotoma | obtusata | 130,000-1000 yBP | New World | North America |
| IUCN | Caniformia | MUSTELIDAE | Neovison | macrodon | 1000 yBP-recent | New World | North America |
| Sandom et al. | Caniformia | URSIDAE | Arctodus | simus | 130,000-1000 yBP | New World | North America |
| Sandom et al. | Caniformia | URSIDAE | Arctotherium | tarijense | 130,000-1000 yBP | New World | South America |
| Sandom et al. | Caniformia | URSIDAE | Arctotherium | wingei | 130,000-1000 yBP | New World | South America |
| Sandom et al. | Caniformia | URSIDAE | Tremarctos | floridanus | 130,000-1000 yBP | New World | North America |
| Sandom et al. | Feliformia | FELIDAE | Homotherium | serum | 130,000-1000 yBP | New World | North America |
| Sandom et al. | Feliformia | FELIDAE | Leopardus | amnicola | 130,000-1000 yBP | New World | North America |
| Sandom et al. | Feliformia | FELIDAE | Miracinonyx | trumani | 130,000-1000 yBP | New World | North America |
| Sandom et al. | Feliformia | FELIDAE | Panthera | atrox | 130,000-1000 yBP | New World | North America |
| Sandom et al. | Feliformia | FELIDAE | Smilodon | fatalis | 130,000-1000 yBP | New World | North and South America |
| Sandom et al. | Feliformia | FELIDAE | Smilodon | populator | 130,000-1000 yBP | New World | South America |
| Sandom et al. | Caniformia | CANIDAE | Cynotherium | sardous | 130,000-1000 yBP | Old World | Sardinia / Corsica |
| Sandom et al. | Caniformia | URSIDAE | Ursus | spelaeus | 130,000-1000 yBP | Old World | Europe |
| IUCN | Feliformia | EUPLERIDAE | Cryptoprocta | spelea | 1000 yBP-recent | Old World | Madagascar |
| Sandom et al. | Feliformia | FELIDAE | Homotherium | latidens | 130,000-1000 yBP | Old World | Europe |
| Sandom et al. | Feliformia | FELIDAE | Panthera | spelaea | 130,000-1000 yBP | Old World | Europe |
|  |  |  |  |  |  |  |  |
| **Source** | **Clade** | **Family** | **Genus** | **Species** | **Extinction** | **Extinct range** | **Current range** |
| Sandom et al. | Caniformia | Canidae | Cuon | alpinus | Regionally extinct | North America + Europe | Asia |
| Sandom et al. | Feliformia | Felidae | Panthera | leo | Regionally extinct | Europe | Africa |
| Sandom et al. | Feliformia | Felidae | Panthera | pardus | Regionally extinct | Europe | Africa + Asia |
| Sandom et al. | Feliformia | Hyaenidae | Crocuta | crocuta | Regionally extinct | Europe + Asia | Africa |

Regionally extinct species of body-mass < 10 kg are not covered, but only very few examples appear to exist [35-38]. IUCN refers IUCNs *Mammals - full taxonomy and Red List status* (<http://www.iucnredlist.org/technical-documents/spatial-data>). Sandom *et al.* (2014) Global Late Quaternary megafauna extinctions linked to humans, not climate change [39]. This table does not include the two extinct species of Pinnipedia.
